# Supplementary material for: Reconciling Mining with the Conservation of Cave Biodiversity: A Quantitative Baseline to Help Establish Conservation Priorities
Source: PLoS One. 2016 Dec 20;11(12):e0168348. doi: 10.1371/journal.pone.0168348 (PMC5173368; doi:10.1371/journal.pone.0168348)
Supplement: S1 Dataset — (ZIP) [file pone.0168348.s002.zip › Taxa/Serra Norte/SN_2013/N4WS/Lista N4WS-21.pdf]

CAVIDADE N4WS-0021

Classe	Ordem	Fam/Outros	Gên/Outros	Espécie	S-U	U-S	Zon.
Arachnida	Araneae	Ctenidae		jovem		1	E
Arachnida	Araneae	Filistatidae		sp.1		X	E
Arachnida	Araneae	Hersiliidae	*Ypypuera*	sp.1		X	E
Arachnida	Araneae	Ochyroceratidae	*Ochyrocera*	sp.1	X		E
Arachnida	Araneae	Ochyroceratidae	*Speocera*	sp.1	X		E
Arachnida	Araneae	Pholcidae	*Leptopholcus*	sp.1		X	E
Arachnida	Araneae	Pholcidae	*Mesabolivar*	*eberhard*		X	E
Arachnida	Araneae	Pholcidae		jovem	X		E
Arachnida	Araneae	Salticidae	*Mago*	sp.1		X	E
Arachnida	Araneae	Scytodidae	*Scytodes*	sp.2	3		E
Arachnida	Araneae	Theraphosidae		jovem		1	E
Arachnida	Araneae	Theridiidae	*Achaearanea*	sp.1		X	E
Arachnida	Araneae	Theridiosomatidae	*Plato*	sp.1	X		E
Diplopoda	Polydesmida	Paradoxosomatidae		sp.1		1	E
Entognatha	Collembola	Entomobryidae		sp.3	X		E
Entognatha	Collembola	Paronellidae		sp.1	X	X	E
Insecta	Blattodea			jovem	4		E
Insecta	Coleoptera	Scarabaeidae		sp.2		X	E
Insecta	Coleoptera	Staphylinidae	Pselaphinae	sp.39	X		E
Insecta	Diptera	Ceratopogonidae		sp.	X		E
Insecta	Diptera	Chloropidae		sp.	X		E
Insecta	Diptera	Dolichopodidae		sp.	X		E
Insecta	Diptera	Tipulidae	Tipulinae	sp.	X		E
Insecta	Heteroptera	Reduviidae	Emesinae	jovem	1		E
Insecta	Heteroptera	Veliidae	*Paravelia*	sp.1		X	E
Insecta	Homoptera	Cixiidae		jovem		X	E
Insecta	Hymenoptera	Formicidae	*Camponotus*	sp.1	X		E
Insecta	Hymenoptera	Formicidae	*Pachycondyla*	*striata*	X		E
Insecta	Hymenoptera	Formicidae	*Solenopsis*	sp.2		X	E
Insecta	Isoptera	Termitidae	*Termes*	sp.	X		E
Insecta	Lepidoptera	Gelechioidea		sp.3		X	E
Insecta	Orthoptera	Phalangopsidae	*Paraclodes*	sp.	3		E
Insecta	Psocoptera	Epipsocidae	*Epipsocus*	sp.2	X		E
Insecta	Thysanura	Nicoletiidae		sp.1	X		E
Malacostraca	Isopoda	Dubioniscidae		sp.1	X		E
Malacostraca	Isopoda	Philosciidae		sp.2		X	E
Symphyla		Scutigerellidae	*Hanseniella*	sp.1		X	E
Mammalia	Chiroptera	Emballonuridae	*Peropteryx*	sp.	1	1	E
Mammalia	Chiroptera	Furipteridae	*Furipterus*	*horrens*	1		E
Reptilia	Squamata	Gekkonidae	*Thecadactylus*	sp.	1		E
